# Attenuation of toxicity and occurrence of degradation products of the fungicide tebuconazole after combined vacuum UV and UVC treatment of drinking water

**DOI:** 10.1007/s11356-022-19691-0

**Published:** 2022-04-02

**Authors:** Oihane Del Puerto, Nuno P. F. Gonçalves, Claudio Medana, Alessandra Bianco Prevot, Peter Roslev

**Affiliations:** 1 Department of Chemistry and Bioscience, Aalborg University, Fredrik Bajers Vej 7H, 9200 Aalborg, Denmark; 2 Dipartimento Di Chimica, Università Di Torino, Torino, Italy; 3 Dipartimento Di Biotechnologie Molecolari E Scienze Della Salute, Università Di Torino, Torino, Italy

**Keywords:** Tebuconazole, 1,2,4-Triazole, Drinking water, Vacuum UV and UVC, Advanced oxidation, Photolysis, Ecotoxicity

## Abstract

Antifungal azoles are the most frequently used fungicides worldwide and occur as active ingredients in many antifungal pharmaceuticals, biocides, and pesticides. Azole fungicides are frequent environmental contaminants and can affect the quality of surface waters, groundwater, and drinking water. This study examined the potential of combined vacuum UV (185 nm) and UVC (254 nm) irradiation (VUV/UVC) of the azole fungicide tebuconazole and the transformation product 1,2,4-trizole on degradation and changes in ecotoxicity. In vivo ecotoxicity was examined before and after UV treatment using bioassays with test organisms from different trophic levels to integrate changes in biological effect of the parent compound and the degradation products. The test battery included the luminescent bacterium *Aliivibrio fischeri*, the Gram-positive bacterium *Bacillus subtilis*, the fungus *Fusarium graminearum*, the green microalga *Raphidocelis subcapitata*, and the crustacean *Daphnia magna*. The combined VUV/UVC treatment of tebuconazole in drinking water efficiently degraded the parent compound at the µg/L-mg/L level and resulted in transformation products with lower toxicity than the parent compound. A direct positive correlation was observed between the applied UV dose (fluence, J/cm^2^), the disappearance of tebuconazole, and the decrease in ecotoxicity. The combined VUV/UVC process does not require addition of supplementary oxidants or catalysts and our study suggests that VUV/UVC-mediated photolysis of azole fungicides in water can decrease the overall toxicity and represent a potentially environmentally friendly treatment method.

**Supplementary Information:**

The online version contains supplementary material available at 10.1007/s11356-022-19691-0.

## Introduction

Antifungal azoles are heterocyclic compounds that inhibit certain pathways of the sterol synthesis, disrupting the structural organization of cell membranes of fungi (Haegler et al. 2017). Azole fungicides are therefore commonly used in biocide products, pesticides, and pharmaceutical drugs to treat fungal diseases. The usage is higher than for any other fungicides worldwide and a significant fraction may not reach the designated target and is sometimes dispersed in the environment by processes such as spray drift, surface runoff, and soil infiltration (Berenzen et al. 2005; Kahle et al. 2008; Chen and Ying 2015; Zhou et al. 2016).

Azoles can be classified into triazoles (three nitrogen atoms) and imidazoles (two nitrogen atoms) according to their chemical structure (Chen and Ying 2015). They are highly lipophilic and moderately persistent with environmental half-lives of weeks to months, and they can remain in the environment long enough to affect non-target organisms (Kahle et al. 2008). Tebuconazole (1-[4-chlorophenyl]-4,4-dimethyl-3-[1H, 1,2,4-triazol-1-ylmethyl]pentan-3-ol), is an active ingredient in triazole fungicides that are widely used in agriculture to control fungal diseases of turf grasses, vegetables, citrus, field crops, and ornamental plants (Katagi 2004). Tebuconazole is mainly applied as a foliar spray but it is also used in seed treatments (Katagi 2004; Danish 2019). Given its high photochemical stability, photodegradation of tebuconazole under natural light is very slow in the environment, and microbial-mediated degradation in soil can take several months (Bending et al. 2007). 1,2,4-triazole [1H-1,2,4-triazole] is an environmental transformation product of several azole fungicides including tebuconazole and has recently been detected as a contaminant in samples of drinking water and groundwater (Danish 2019; Rosenbom et al. 2021).

Triazole fungicides and their transformation products are known to affect non-target organisms and several studies have highlighted potential adverse effects at different trophic levels including bacteria, microalgae, fungi, invertebrates, and fish (Andreu-Sánchez et al. 2011; Sun et al. 2014; Li et al. 2015; Sancho et al. 2016; Westlund et al. 2018). In addition, tebuconazole has been classified as a possible human carcinogen and has the potential to disrupt the endocrine system (Yu et al. 2013; Zhou et al. 2016). However, the ecotoxicological impacts of tebuconazole transformation products such as 1,2,4-triazole are less known. Azole fungicides and their degradation products have now emerged as an important class of aquatic pollutants and environmentally friendly methods for their removal are in demand.

UV-based technology in combination with advanced oxidation processes (AOPs) has been suggested as promising tools for removal of organic contaminants from water (Braun et al. 2004; Moussavi et al. 2014; Zoschke et al. 2014; Rozas et al. 2016; Ye et al. 2019). For example, UVC light (254 nm) has been combined with hydrogen peroxide (H_2_O_2_) to enhance photolysis and different photocatalysts including semiconductors such as titanium dioxide (TiO_2_) and zinc oxide (ZnO) have been employed for removal of organic micropollutants from drinking water and wastewater (Kamat et al. 2002; Thiruvenkatachari et al. 2008; Ijpelaar et al. 2010; Moussavi et al. 2014; Rozas et al. 2016; Mecha et al. 2017). However, the operational costs and potential inconvenience of procedures that require addition and regeneration of catalysts have resulted in an interest in alternative cost-efficient UV technologies. In this context, a combination of UVC and vacuum UV (185 nm) irradiation (VUV/UVC) has attracted attention as a highly efficient technology for degradation of organic contaminants in aqueous solutions without the need for addition of oxidants or catalysts (Braun et al. 2004; Zoschke et al. 2014; Pan et al. 2022). Low-pressure mercury lamps can emit light at both 185 nm and 254 nm and the process results in generation in situ of strong oxidants such as hydroxyl radical (^•^OH), superoxide anion (^•^O_2_^−^), and hydrogen peroxide (H_2_O_2_) due to the photolysis of water at high irradiation intensities. The VUV/UVC process offers an advantage compared to UVC photocatalysis as the former process does not require addition of supplementary oxidants or catalysts for efficient degradation. Thus, several studies have explored the potential for degradation of organic trace compounds in water by VUV and VUV/UVC irradiation, and some studies have also included mechanistic and kinetic reactions of VUV-irradiated aqueous pesticides (Imoberdorf and Mohseni 2011b; Zoschke et al. 2014; Duca et al. 2017; Ye et al. 2019). However, relatively few studies have included toxicological analyses of VUV-treated aqueous pesticides and transformation products. This is relevant because degradation of parent molecules does not necessarily imply complete mineralization of the parent compound and attention needs to be paid to the potential harmful effects of transformation products and occurrence of reactive species in the treated water.

The current study investigated the degradation kinetics, occurrence of transformation products, and ecotoxicity changes of tebuconazole and 1,2,4-triazole after combined VUV/UVC irradiation in drinking water. A specific aim was to determine to what degree VUV/UVC irradiation could facilitate direct and/or indirect photolysis and thereby attenuate toxicity to different organisms. Direct photolysis involves light absorption by the target compound with subsequent transformation whereas indirect photolysis can be described as degradation mediated by reactive species generated during the process. Changes in toxic response were compared before and after VUV/UVC irradiation using a battery of test organisms that included *Aliivibrio fischeri*, *Bacillus subtilis*, *Raphidocelis subcapitata*, *Fusarium graminearum*, and *Daphnia magna.* Organisms from different trophic levels were included to better assess biological effects of all bioactive compounds in the samples after VUV/UVC treatment including transformation products and reactive species.

## Materials and methods

### Chemicals

The triazole fungicide tebuconazole (1-[4-chlorophenyl]-4,4-dimethyl-3-[1H, 1,2,4-triazol-1-ylmethyl]pentan-3-ol) (CAS 107534–96-3; > 98% purity) and the degradation product 1,2,4-triazole (1H-1,2,4-triazole) (CAS 288–88-0; > 98% purity) were obtained from TCI Europe (Belgium). These compounds are hereafter referred to as TEB and 124T, respectively. Stock solutions of TEB and 124T were prepared in autoclaved distilled deionized water and stored in the dark at 5 °C.

### Drinking water

Drinking water was collected at Aalborg Municipality (Denmark). The source water in Aalborg Municipality is hard groundwater (12°dH) abstracted directly from chalk aquifers. No water treatment or disinfection is employed by the municipality before distribution of the drinking water to the consumers. The drinking water is naturally nutrient poor with a concentration of non-volatile organic carbon (NVOC), NO_3_^−^, NH_3_/NH_4_^+^, NO_2_^−^, and total P of 0.99 mg/L, 1.3 mg/L, 0.007 mg/L, < 0.001 mg/L, and 0.01 mg/L, respectively. The water temperature, pH, and turbidity were 8.8 °C, 7.6, and < 0.13 FTU, respectively. The background concentrations of TEB and 124T in the drinking water were below the level of detection (< 0.01 µg/L).

### Vacuum UV irradiation

**Fig. 1:**
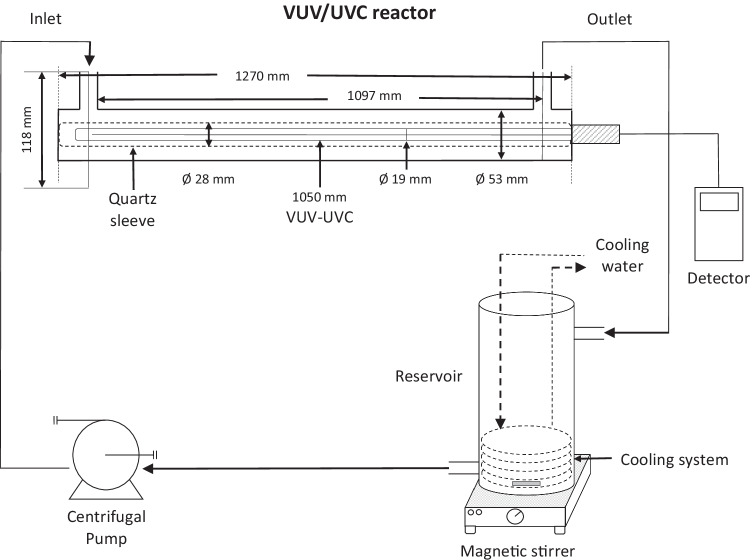
Schematic representation of the photochemical VUV/UVC reactor system

The effect of combined vacuum UV and UVC irradiation of TEB in drinking water was investigated in a continuous-flow UV photoreactor (ULTRAAQUA A/S, Aalborg, Denmark). The VUV photoreactor consisted of a tubular stainless steel reactor with an inner diameter of 53 mm, a length of 1270 mm, and a reactor volume of 1.7 L (Fig. 1). The photoreactor was connected to a 2.3 L stainless steel reservoir and a diaphragm pump operated at 2 L/min with recirculation (Siebec, pompe M7). The reservoir was equipped with a magnetic stir bar to facilitate mixing, and a stainless steel cooling spiral operated at 10 °C to prevent heating.

The UV photoreactor was equipped with a low-pressure high-output amalgam VUV Hg lamp with a 1050 mm length and a 19 mm diameter (UltraTherm 200 W LPHO TOC UV, Ultraaqua A/S, Denmark). The UV lamp simultaneously emitted VUV (185 nm) and UVC (254 nm) at a radiation flux of 14 W and 56 W, respectively (1:4 ratio). The VUV/UVC lamp was located inside a high-purity 28-mm-diameter quartz tube transparent to both wavelengths. The theoretical thickness of the water film around the quartz sheath was 12.5 mm. The reactor was equipped with an UVC sensor and the maximum irradiance in drinking water was 340 W/m^2^ for UVC corresponding to 85 W/m^2^ of VUV.

VUV irradiation experiments were conducted by loading the photoreactor and reservoir with a total of 4 L of drinking water spiked with TEB or 124T with different nominal concentrations (1 µg/L to 12 mg/L and 0.1–120 mg/L, respectively). The water was recirculated for 64 min and 10 mL samples were collected at the outlet of the photoreactor before turning on the VUV lamp (0 min) and at 1, 2, 4, 8, 16, 32, and 64 min after turning on the VUV lamp corresponding to combined VUV/UVC doses (fluence) of 0, 0.78, 1.56, 3.12, 6.25, 12.5, 25, and 50 J/cm^2^.

### Detection of reactive oxygen species

The presence of reactive oxygen species such as hydroxyl radicals (^•^OH) in aqueous samples during VUV/UVC irradiation was confirmed by the addition of a radical probe to the feed water. The aromatic molecule 2H-Chromen-2-one (coumarin) is converted to the highly fluorescent *7-*hydroxycoumarin when exposed to hydroxyl radicals (^•^OH). Radicals were detected as fluorescence after addition of 1 mM coumarin during VUV/UVC treatment (CAS 91–64-5; Merck, Denmark). Fluorescence originating from radical reaction were quantified after 0, 1, 2, 4, 8, 16, 32, and 64 min using a Victor X2 Multilabel Plate Reader (Perkin Elmer).

### Degradation of TEB and occurrence of transformation products

A Summit Dionex Corporation HPLC with UV detection at 220 nm was used to quantify the concentration of TEB in water samples after VUV/UVC degradation. The HLPC was equipped with a Luna 5μ C18 100 Å column (250 × 4.60 mm), and acetonitrile/water (50:50 v:v) was used as mobile phase at a flow of 1 mL/min.

Non-target liquid chromatography–high-resolution mass spectrometry analysis (LC-HRMS) of water samples with TEB was performed before and after VUV/UVC exposure to identify transformation products. Analyses were carried out using an Ultimate 3000 High-Pressure Liquid Chromatography coupled through an ESI source to an LTQ-Orbitrap mass spectrometer (Thermo Scientific). Chromatographic separation was achieved using a reversed-phase C18 column (Phenomenex Luna, 150 × 2 mm, 3 µm, 110 Å; Phenomenex, Italy) by injecting a 10 µL sample volume at a mobile phase consisted of a mixture of 0.1 mM formic acid (eluent A) and acetonitrile (eluent B). The gradient profile started with 5% B, increased up to 100% B in 40 min, and to 100% A in 10 min. Samples were ionized in both positive and negative ionization modes. The LC effluent was delivered to the ESI ion source using nitrogen as sheath and auxiliary gas with the following parameters: sheath gas 34 arbitrary unit (arb), auxiliary gas 15 arb, capillary voltage 4.48 kV, and capillary temperature of 270 °C. Full mass spectra were acquired in positive ion mode with a resolution of 30,000. Data analysis was performed using the MZmine 2.53 for peak alignment, peak grouping, background noise, and retention time correction, and the METLIN database was used to identify the transformation products.

#### Toxicity test with the luminescent bacterium *Aliivibrio fischeri.*

Toxicity screening of samples with TEB and 124T were examined in standard inhibition tests with the luminescent bacterium *Aliivibrio fischeri* (ISO 11348–1 2007). *A. fischeri* DSM 7151 was incubated in white 96-well plates (CulturPlate, Perkin Elmer) with serial twofold dilutions of TEB resulting in 10 nominal concentrations and 8 replicates. Changes in bioluminescence was quantified after 30 min using a Victor X2 Multilabel Plate Reader (Perkin Elmer). The toxicity of TEB to *A. fischeri* was examined before and after exposure of aqueous solutions to different VUV/UVC irradiation regimes.

#### Toxicity test with the bacterium *Bacillus subtilis.*

Toxicity of TEB and 124T to Gram-positive bacteria was examined in a newly developed inhibition test with *Bacillus subtilis* DSM 10 (Papagiannaki et al. 2020). The endpoint in this test was inhibition of growth and hydrolase activity after 18 h of bacterial growth in microplates at 30 °C (Papagiannaki et al. 2020). The toxicity of TEB to *B. subtilis* was examined before and after exposure of aqueous solutions to different VUV/UVC irradiation regimes.

#### Toxicity test with the fungus *Fusarium graminearum* .

The toxicity of TEB and 124T to a filamentous fungus was examined in an inhibition test with the plant pathogen *Fusarium graminearum*. We used this organism to develop a Fusarium Toxicity assay (FUTOX) in which the endpoint was inhibition of chitinolytic enzyme activity after growth for 72 h. *F. graminearum* was cultivated at 25  C in a Fusarium Minimal Medium (FMM) with the following composition (g/L): 0.12 Na_2_SO_4_, 0.05 MgSO_4_*7H_2_O, 0.008 CaCl_2_*2H_2_O, 0.268 NH_4_Cl, 5.0 KNO_3_, 1.14 Na_2_HPO_2_, 0.272 KH_2_PO_4_, 0.5 yeast extract, 1.0 proteose peptone, 10.0 glucose, and 10.0 maltose. The medium was supplemented with the following trace elements (mg/L): 1.39 FeSO_4_*7H_2_O, 0.054 ZnCl_2_, 0.068 CuCl_2_*2H_2_O, 0.021 NaBr, 0.024 Na_2_MoO_2_*2H_2_O, 0.079 MnCl_2_*4H_2_O, 0.033 KI, 0.025 H_3_Bo_3_, 0.048 CoCl_2_*6H_2_O, and 0.048 NiCl_2_*6H_2_O. Prior to the FUTOX test, *F. graminearum* was grown for 7–10 days at 25  C on agar plates consisting of the above FMM supplemented with 1.8 g/L agar.

TEB and 124T was diluted twofold in 96-well black microplates (CulturPlate, Perkin Elmer) by serially diluting 150 µL of an aqueous solution with the chemical in 150 µL FMM medium. After transfer of the chemicals, 150 µL of diluted *F. graminearum* culture was added to each well resulting in a final liquid volume of 300 µL in each well. The diluted *F. graminearum* culture used as inoculum in the FUTOX assay consisted of conidia harvested from FMM agar plates and resuspended in FMM to a density of *A*_600_ = 0.01. Sealed microplates were incubated without shaking for 72 h ± 2 h at 25 °C. The activity of the hydrolytic enzyme chitinase in *F. graminearum* (1 → 4)-2-acetamido-2-deoxy-β-D-glucan glycanohydrolase) was measured by adding 30 µL of the fluorescent substrate 4-methylumbelliferyl N-acetyl-β-D-glucosaminide (4-methylumbelliferyl 2-acetamido-2-deoxy-β-D-glucopyranoside). The fluorescent chitinase substrate was added to each well from a concentrated stock solution in dimethyl sulfoxide to obtain a final concentration of 10 µM. After 120-min incubation at 25  C, fluorescence was quantified in each well using a Victor X2 Multilabel Plate Reader with a 355 nm excitation and 460 nm emission filter (Perkin Elmer). The bioassay with *F. graminearum* included eight replicates of blanks (medium only), controls (no test chemical), and 10 nominal concentrations of TEB and 124T. The toxicity of TEB to *F. graminearum* was examined before and after exposure of aqueous solutions to different VUV/UVC irradiation regimes.

#### Toxicity test with the green microalga *Raphidocelis subcapitata.*

The toxicity of TEB and 124T to phytoplankton was examined in inhibition tests with the unicellular green microalgae *Raphidocelis subcapitata* (formerly *Selenastrum capricornutum* and *Pseudokirchneriella subcapitata*). The endpoint was inhibition of growth measured after 72 h of incubation as described in ISO 8692 (2012). *R. subcapitata* (MicroBioTests Inc.) was cultivated in alga test medium at 22 ± 2 °C and continuous illumination at 6500 lx (ISO 8692, 2012). Two-fold dilutions of TEB or 124T were prepared in 96-well clear Nunclon microplates (Thermo Scientific) by serially diluting 150 µL of an aqueous solution with the chemical in 150 µL algal test medium. After transfer of the chemicals, 150 µL of diluted *R. subcapitata* culture (1:50) was added to each well resulting in a final liquid volume of 300 µL in each well. Plates were incubated for 72 h at 22 ± 2 °C on a shaker at 70 rpm with continuous illumination (6500 lx). Growth was measured after 0, 24 h, 48 h, and 72 h as absorbance at 450 nm using a Thermo Multiskan Plate Reader (Thermo Scientific). The bioassay with *R. subcapitata* included eight replicates of blanks (medium only), controls (no test chemical), and 10 nominal concentrations of TEB and 124T. The toxicity of TEB to *R. subcapitata* was examined before and after exposure of aqueous solutions to different VUV/UVC irradiation regimes.

#### Toxicity test with the crustacean *Daphnia magna.*

The toxicity of TEB and 124T to zooplankton was examined in inhibition tests with the crustacean *D. magna* (ISO 6341, 2012). The toxicological endpoint was inhibition of mobility determined by visual inspection of the animals (ISO 6341, 2012). *D. magna* STRAUS was cultivated from a laboratory clone originating from pure-culture ephippia (MicroBioTests Inc.). Each treatment consisted of 20 juvenile animals distributed among 4 glass vials with 5 animals and 10 mL freshwater medium in each 30 mL vial. The mobility of each animal was determined after 24 h and 48 h (ISO 6341, 2012). The toxicity of TEB to *D. magna* was examined before and after exposure of aqueous solutions to different VUV/UVC irradiation regimes.

### Data analysis and statistics

The toxic responses measured for all endpoints were expressed as inhibition (*I*) relative to control samples: *I* = 1 − (*R*_*i*_ / *R*_*c*_), where *R*_*i*_ and *R*_*c*_ are the responses measured for inhibited and control samples, respectively. Control samples included samples without VUV/UVC irradiation and TEB or 124T, and water samples without TEB or 124T but with VUV/UVC irradiation to assess any toxicity associated with reactive oxygen species generated during irradiation of aqueous solutions. Concentration–response curves were fitted to a Log-logistic model using iterative non-linear regression:

$$\mathrm{Response}=A1+\frac{A2-A1}{1+{10}^{\left(\left(\mathrm{Log} X - C\right)p\right)}}$$

where *A*_1_ is the bottom asymptote, *A*_2_ is the top asymptote, *X* refers to the median effective concentration (EC50), *C* is the toxicant concentration (mg/L), and *p* is a model parameter representing the slope of the curve. Iterative non-linear regressions and calculation of 95% confidence limits for EC50 values were performed using OriginPro (OriginPro 2021).

Statistical comparisons of results were carried out using the non-parametric Kruskal–Wallis *H* test for evaluating differences among multiple treatments, and the Mann–Whitney *U* test (Wilcoxon rank sum test) was used for evaluating differences between controls and defined treatments. Statistical analyses were carried out using KaleidaGraph 4.5.4 (Synergy Software, USA) with a significance level of *p* < 0.05.

## Results and discussion

### Degradation kinetics and transformation products

**Fig. 2:**
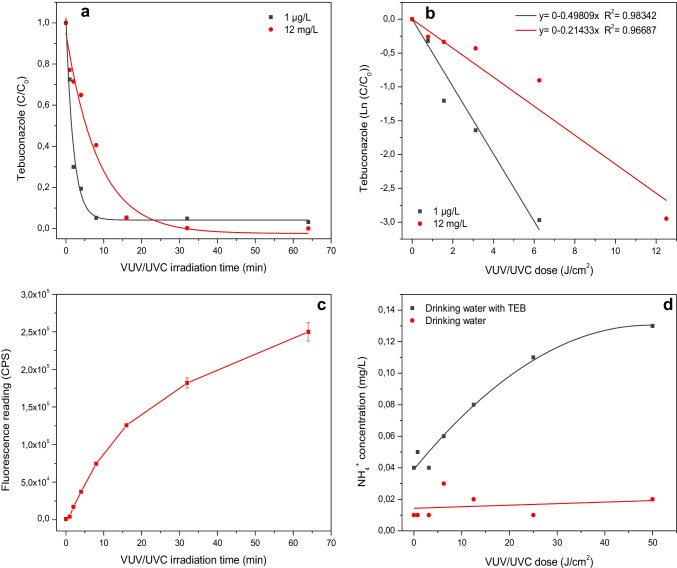
VUV/UVC-mediated degradation of aqueous TEB as a function of irradiation time (**a**) and VUV/UVC dose during the initial 8 min (**b**), detection of active oxygen species as fluorescence using coumarin as probe (**c**), and ammonium levels in drinking water with and without 12 mg/L TEB during VUV/UVC irradiation (**d**)

The degradation kinetics of TEB during VUV/UVC irradiation was analyzed over time and VUV/UVC dose (Fig. 2a, b). TEB degradation was faster at trace levels (1 µg/L) compared to elevated concentrations (12 mg/L), and 95% and 60% of the parent compound were removed after 8 min of VUV/UVC irradiation (6.25 J/cm^2^), respectively (Fig. 2a, b). The initial degradation of TEB followed pseudo-first-order kinetics (Fig. 2b), and the first-order rate coefficient was about 2 times greater at 1 µg/L (*k* = 0.39 min^−1^ ≈ *k* = 0.50 [J/cm]^−1^) than at 12 mg/L (*k* = 0.17 min^−1^ ≈ *k* = 0.21 [J/cm]^−1^). The first-order rate coefficients corresponded to initial half-lives (*T*_50_) of 1.8 min and 4.1 min for 1 µg/L TEB and 12 mg/L TEB, respectively. These estimates support the observation that removal of azole fungicide by combined VUV/UVC is concentration dependent and is more efficient at lower concentrations (µg/L) which has also been shown for an antifungal pharmaceutical (Gonçalves et al. 2021).

The pseudo-first-order kinetics for TEB removal (Fig. 2b) was supported by measurements of reactive oxygen species during VUV/UVC irradiation (Fig. 2c). The low-pressure Hg lamp used in the current study simultaneously emits polychromatic radiation at 185 nm wavelength and 254 nm wavelength and will subsequently induced photolysis of water and generate very reactive and non-selective species (e.g., ^•^OH, ^•^H, ^•^HO_2_, ^•^O_2_^−^, e^−^_aq_) (Gonzalez et al. 2004; Imoberdorf and Mohseni 2011a; Zoschke et al. 2014). UVC at 254 nm has a deeper penetration in water than VUV irradiation at 185 nm but the latter irradiation type has a greater potential for generation of reactive species. The aromatic molecule 2H-Chromen-2-one (coumarin) was used as a probe for reactive species and coumarin was converted to the highly fluorescent *7-*hydroxycoumarin when exposed to hydroxyl radicals (^•^OH). Reaction with the hydroxyl radical probe occurred throughout the VUV/UVC incubation but appeared relatively greater in the initial phase of the VUV/UVC irradiation period (Fig. 2c).

Ion chromatography analysis of inorganic nitrogen showed an increase in ammonium concentrations in drinking water with TEB during VUV/UVC irradiation (Fig. 2d). Ammonium concentrations in aqueous TEB samples increased with VUV/UVC irradiation time compared to ammonium levels in drinking water without TEB, suggesting that part of the triazole moiety of TEB had been mineralized to ammonium (Fig. 2d). The release of ammonium has also been suggested for other degradation pathways involving triazoles and may involve both oxidized and reduces nitrogen species (Ghanbari et al. 2020; Yang et al. 2021).

### Identification of TEB transformation products

**Fig. 3:**
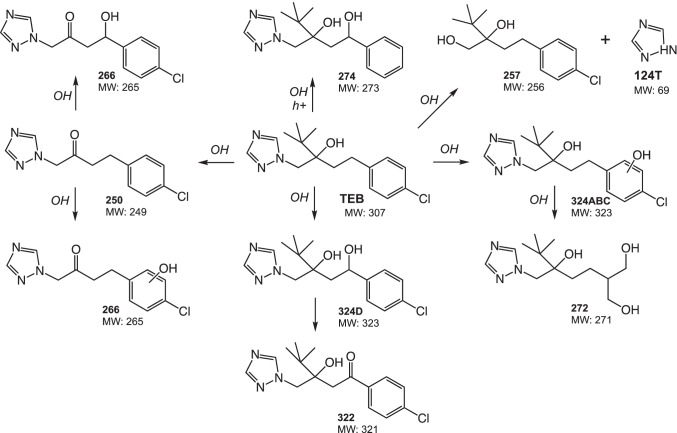
Proposed degradation pathways for aqueous tebuconazole during combined VUV/UVC irradiation

The occurrence of transformation products (TPs) after combined VUV/UVC irradiation of TEB in drinking water was analyzed by liquid chromatography–high-resolution mass spectrometry (LC-HRMS). TEB can absorb UVC irradiation and direct photolysis is therefore possible and VUV irradiation induces homolysis and photochemical ionization of water, thereby generating reactive species resulting in indirect photolysis (Moussavi et al. 2014). A total of 12 TPs were identified which were mainly formed from the addition of the hydroxyl group in different position of the TEB molecule (C_16_H_23_ClN_3_O), the loss of the imidazole group, and subsequent chemical transformations (Table 1; Fig. 3). The identified species here have also been observed during photocatalytic degradation of TEB and structurally elucidated by means of HRMS (Calza et al. 2002; Stamatis et al. 2015).

**Table 1:**
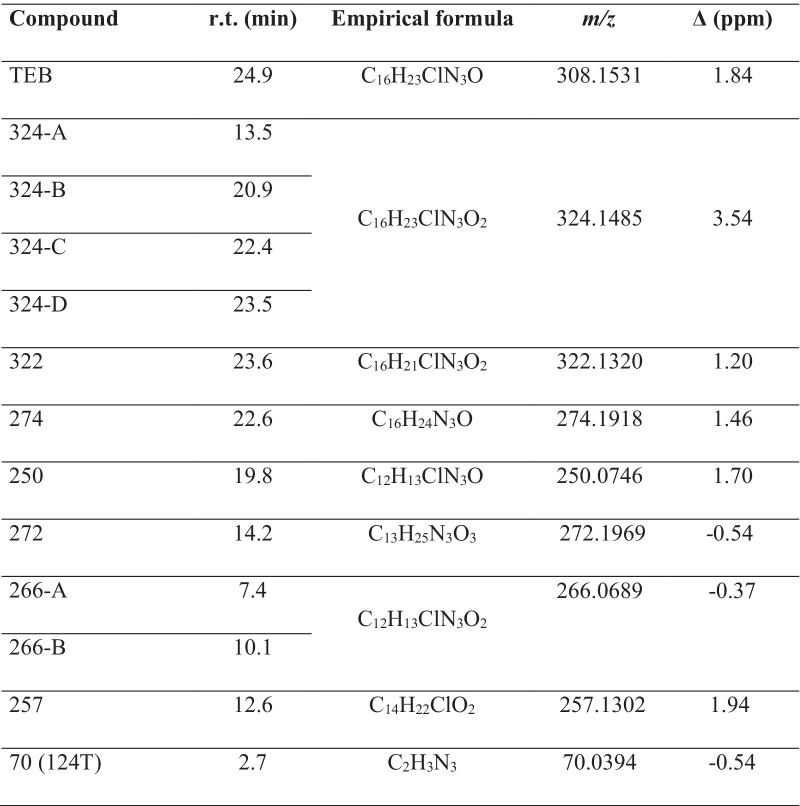
Transformation products identified by LC-HRMS after VUV/UVC irradiation of TEB in drinking water

The degradation profile and occurrence of TPs over time showed that most species appeared at their highest concentrations during the initial 4 min of VUV/UVC irradiation (VUV/UVC dose of 3.12 J/cm^2^) (Supplementary material Fig. S1). However, three TPs were detected at the highest concentration after exposure to extended VUV/UVC dose for 32 min of irradiation corresponding to 25 J/cm^2^ (Supplementary material Fig. S1). Among the identified TPs, four isomers with *m*/*z* 324 were formed matching with the formulae C_16_H_23_ClN_3_O_2_ attributed to the mono-hydroxyl group addition in the aromatic ring (324A, B, and C), and in the adjacent carbon to the phenyl ring (324D) (Table 1). These results correspond to degradation mechanism observed in the presence of the photocatalyst TiO_2_ and the isobaric species were attributed based on the relative retention times (Calza et al. 2002; Stamatis et al. 2015). The oxidation of the last isomer (324D) resulted in a molecule having *m*/*z* 322 with a retention time slightly higher with respect to the formed compound (Table 1). Further OH substitution in the aromatic ring of the remaining isomers led to the formation of the compound at *m*/*z* 272 (Fig. 3). Another specie at *m*/*z* 250 was attributed to the loss of the butyl group probably by the C–C bond cleavage from the OH attack followed by the oxidation of the alcohol group. Additional OH group addition in different position led to the formation of two species at *m*/*z* 266, and a specie at *m*/*z* 274 matching with the formulae C_16_H_24_N_3_O was attributed to the dechlorinated derivative with hydroxyl group addition. Finally, two species were also formed from the typical loss of the triazole moiety (*m*/*z* 70) generating the compound *m*/*z* 257 and *m*/*z* 70 corresponding to the known TEB degradation product 124T. The triazole 124T has been reported to be a major transformation product of azole fungicides during UVB and UVC irradiation in photolytic experiments (Da silva et al. 2001). 124T may also occur naturally in groundwater and soil and has been linked to environmental degradation of tebuconazole and related fungicides (Albers et al. 2021; Rosenbom et al. 2021).

### Toxicity of TEB and 124T to aquatic organisms

**Fig. 4:**
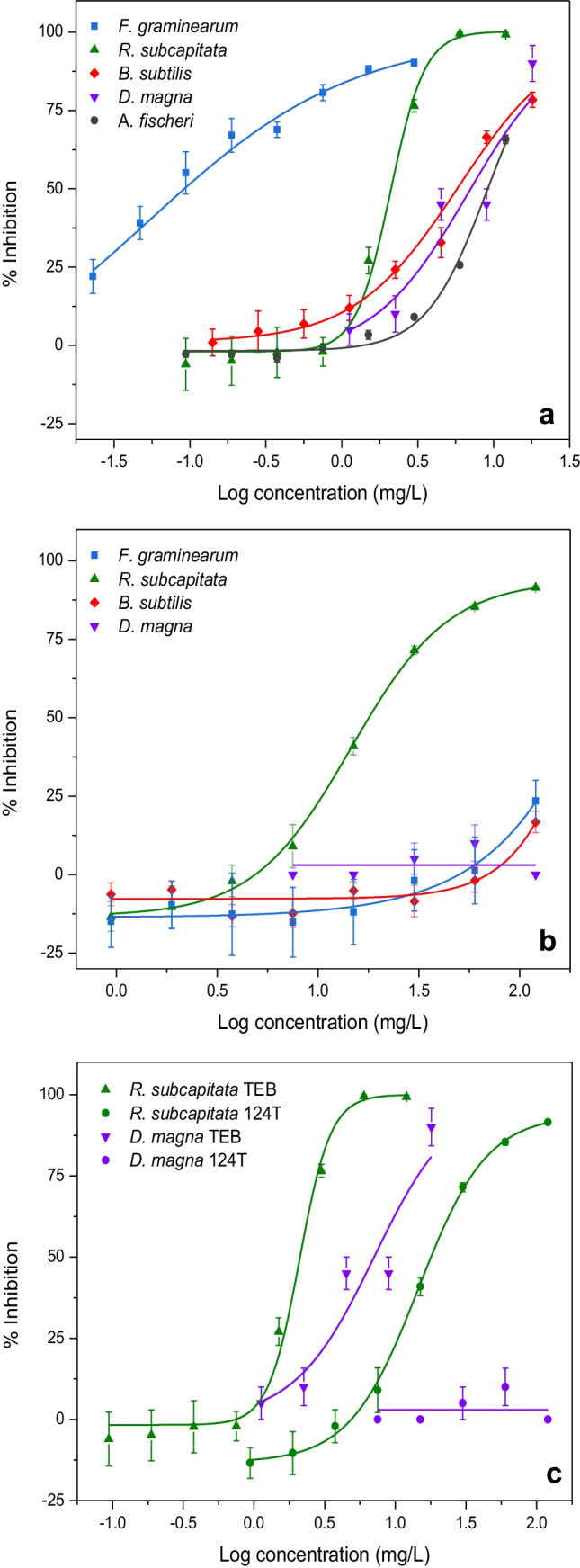
Toxicity of TEB (**a**) and 124T (**b**) to a battery of aquatic organisms that included *F. graminearum*, *R. subcapitata*, *B. subtilis*, *D. magna*, and *A. fischeri*. **c** The toxicity response of *R. subcapitata* and *D. magna* to TEB and 124T. All data points represent mean ± standard error

The in vivo toxicity of TEB and the transformation product 124T to different organisms was tested initially to identify responsive bioassays for further experiments (Fig. 4). The fungus *F. graminearum* was the most responsive organisms to TEB with a median effective concentration (EC50) of 0.05 mg/L, followed by *R. subcapitata*, *B. subtilis*, and *D. magna* with EC50 values of 2.09, 5.56, and 6.75 mg/L, respectively (Table 2). The high susceptibility of *F. graminearum* to TEB is in-line with previous studies reporting EC50 values in the same range although strain variations may occur with occasional higher values (Spolti et al. 2012; Sun et al. 2014). The estimated EC50 values for *R. subcapitata*, *B. subtilis*, and *D. magna* were also in the same range as reported in the literature (Guo et al. 2019). Interestingly, the luminescent bacterium *A. fischeri* frequently used for toxicity screening (ISO 11348–1 2007) was the least responsive test organisms with EC50 value of 9.00 mg/L for TEB (Fig. 4; Table 2). A comparable result has been obtained in a related study reporting EC50 values of 12.07 mg/L for *A. fischeri* exposed to TEB (Westlund et al. 2018). Hence, *A. fischeri* was only included in selected subsequent toxicity tests and *F. graminearum*, *R. subcapitata*, *B. subtilis*, and *D. magna* were used as primary test organisms.

**Table 2:**
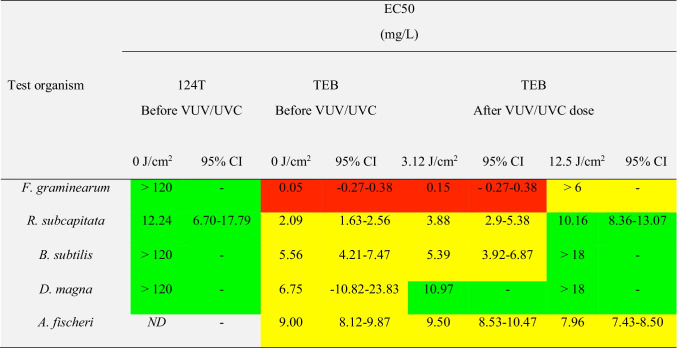
EC50 values of test organisms before and after VUV/UVC irradiated aqueous TEB and 124T

The short-term toxicity of hazardous substances to aquatic organisms can be classified based on LC50/EC50 values (United Nations, 2021). Red color indicates EC50 ≤ 1 mg/L, yellow color indicates 1 mg/L < EC50 ≤ 10 mg/L, and green color indicates EC50 > 10 mg/L

*CI* confidence interval, *ND* not determined

The in vivo toxicity of the transformation product 124T was lower than that of the parent compound TEB (Fig. 4c; Table 2). The EC50 value for 124T was 6 times lower than that of TEB when *R. subcapitata* was used as test organisms (EC50 value of 12.24 and 2.09 mg/L, respectively). 124T was even less toxic to the filamentous fungus *F. graminearum*, the bacterium *B. subtilis*, and the aquatic crustacean *D. magna* with apparent EC50 values > 120 mg/L (Fig. 4; Table 2). No significant toxicity could be detected for *D. magna* exposed to 124T even at the highest test concentration of 120 mg/L (Kruskal–Wallis, *p* = 0.176). This in agreement with registration dossier from the European Chemical Agency indicating that 124T is not particularly harmful to many aquatic organisms due to LC50/EC50 values >100 mg/L (ECHA, 2021).

### Effect of VUV/UVC irradiation of aqueous TEB on test organisms

**Fig. 5:**
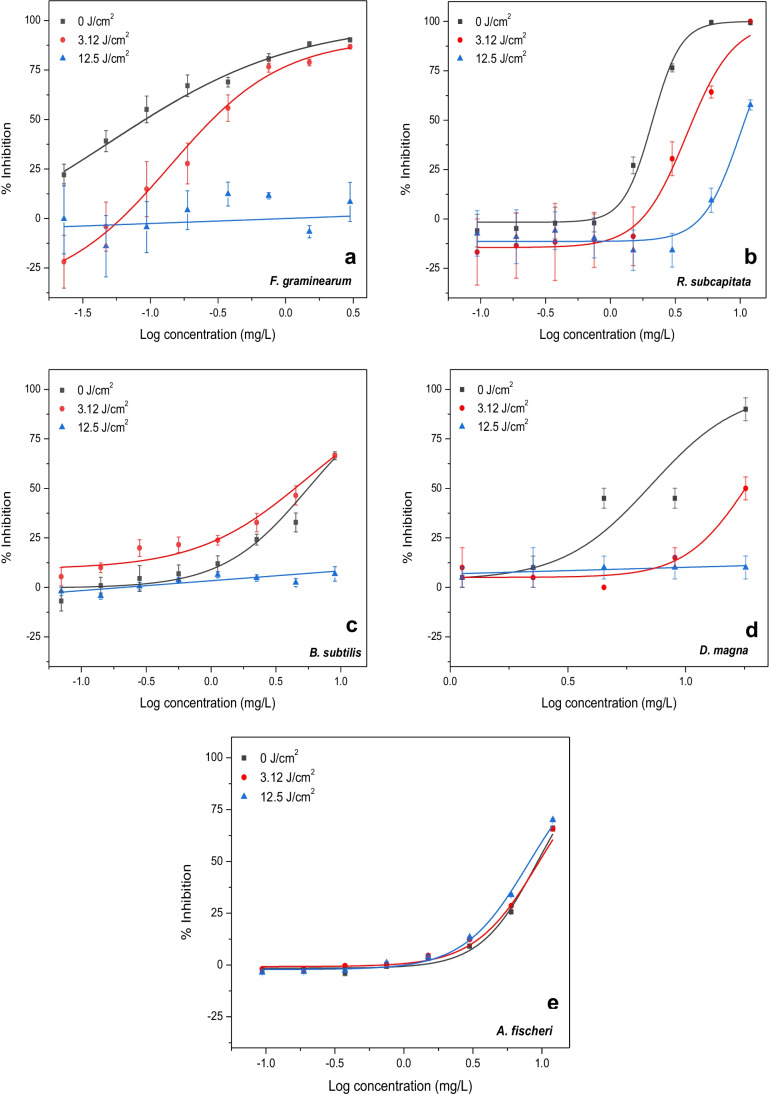
Toxicity before (0 J/cm^2^) and after VUV/UVC irradiation of tebuconazole in drinking water (3.12- and 12.5-J/cm^2^ dose) to **a** *F. graminearum*, **b** *R. subcapitata*, **c** *B. subtilis*, **d** *D. magna*, and **e** *A. fischeri*. Data points represent mean ± standard error

The ability of VUV/UVC treatment to mitigate the toxicity of TEB was assessed in bioassays with *F. graminearum*, *R. subcapitata*, *B. subtilis*, *D. magna*, and *A. fischeri* (Fig. 5). TEB in drinking water was exposed to combined VUV/UVC irradiation in a continuous flow through reactor (Fig. 1), and samples were collected after 4 min and 16 min corresponding to combined VUV/UVC doses of 3.12 J/cm^2^ and 12.5 J/cm^2^, respectively. The selected sampling times were chosen based on occurrence of TPs at the highest relative concentration at 3.12 J/cm^2^ and almost complete removal of TEB at 12.5 J/cm^2^ (Fig. 2; Supplementary material Fig. S1). The combined VUV/UVC treatment of aqueous TEB had a considerable mitigating effect on the toxicity to all test organisms except *A. fischeri* (Fig. 5; Table 2). The short-term (acute) toxicity of hazardous substances to aquatic organisms may be classified into different categories based on LC50/EC50 values and these categories are indicated in Table 2 (United Nations 2021). The toxicity of TEB to *F. graminearum* decreased noticeable and the EC50 value increased threefold after 3.12 J/cm^2^ of VUV/UVC irradiation (Fig. 5a; Table 2). A significant toxic response to *F. graminearum* was no longer detectable after 12.5-J/cm^2^ VUV/UVC irradiation of TEB (Kruskal–Wallis, *p* = 0.139). Similarly, it was no longer possible to determine a median effective concentration for *B. subtilis* after 12.5 J/cm^2^ of VUV/UVC irradiation (Fig. 5c; Table 2). The toxicity of TEB to *R. subcapitata* was attenuated twofold after 3.12 J/cm^2^ of VUV/UVC and fivefold after 12.5 J/cm^2^ (Fig. 5b; Table 2). In the case of *D. magna*, the EC50 value increased from 6.75 to 10.97 mg/L after 3.12 J/cm^2^ of VUV/UVC irradiation whereas the higher VUV/UVC irradiation of 12.5 J/cm^2^ alleviated toxicity considerably and a significant toxic response to *D. magna* was no longer detectable (Kruskal–Wallis, *p* = 0.956). No EC50 could therefore be estimated (Fig. 5d; Table 2). *A. fischeri* was the least responsive organism to aqueous TEB before VUV/UVC treatment and the toxicity did not differ much after VUV/UVC irradiation of TEB (Fig. 5e). No significant difference in toxicity of TEB to *A. fischeri* was observed before and after VUV/UVC treatment (Man-Whitney; *p* > 0.253). As a result, the EC50 values for this organism were comparable before and after VUV/UVC treatment of TEB in drinking water (Table 2). In contrast, it was not possible to determine median effective concentrations for *F. graminearum*, *B. subtilis*, and *D. magna* after 12.5 J/cm^2^ of VUV/UVC irradiation of TEB because the apparent EC50 had increased beyond the maximum nominal test concentration (Table 2).

**Fig. 6:**
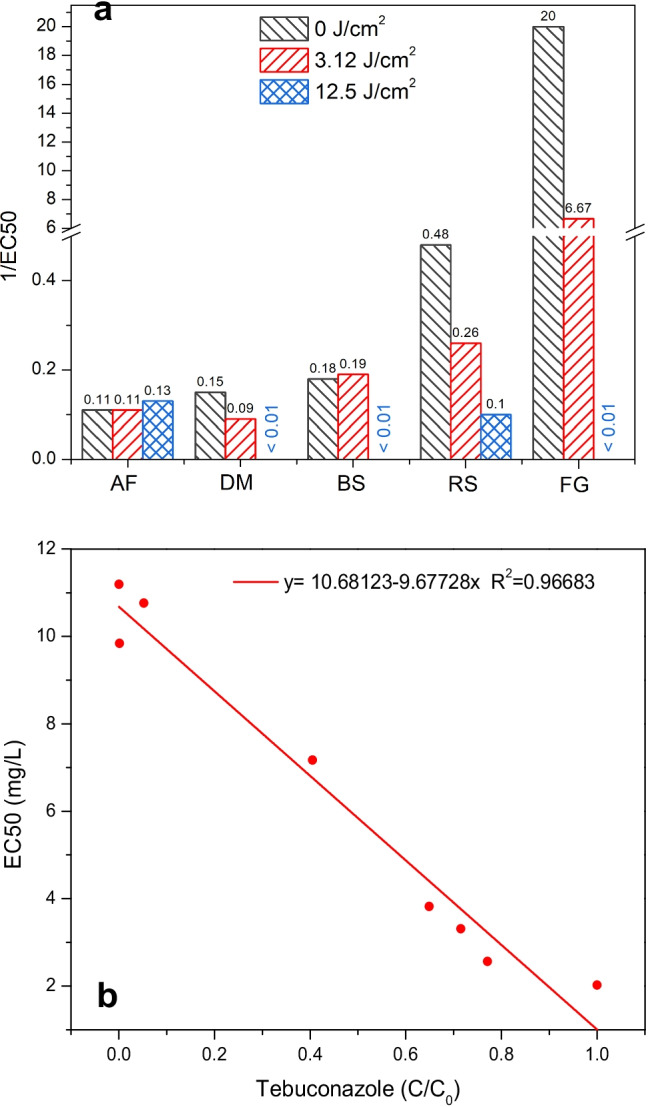
Decreases in toxicity of tebuconazole to different test organisms expressed as 1/EC50 values after exposure to VUV/UVC irradiation (**a**). The test battery included *A. fischeri* (AF), *D. magna* (DM), *B. subtilis (*BS), *R. subcapitata* (RS), and *F. graminearum* (FG). Relationship between degradation of tebuconazole after VUV/UVC irradiation and EC50 values for *R. subcapitata* (**b**)

The ability of combined VUV/UVC treatment to attenuate the toxicity of TEB to all test organisms can be illustrated by the relationship between the 1/EC50 values and the VUV/UVC dose (Fig. 6a). Decreases in toxicity after VUV/UVC irradiation resulted in decreasing 1/EC50 values and were observed for all test organisms except *A. fischeri* (Fig. 6a). Figure 6b illustrates this point further by displaying the relationship between the median effective concentration for VUV/UVC-treated samples with TEB and the degradation of TEB measured during a continuous VUV/UVC irradiation for 64 min (*C*/*C*_0_). It appears that VUV/UVC was not only able not to efficiently degrade TEB but also attenuate the toxicity proportionally (Fig. 6b). Hence, the continuous increase in EC50 values with increasing VUV/UVC irradiation and TEB degradation suggested that the transformation products formed by the VUV-UVC-mediated photolysis were less toxic than the parent compound. These results corroborate the concept that toxicity bioassays can complement chemical analyses by providing additional information about the presence of bioactive compounds in water samples after treatment (Escher and Leusch 2012).

## Conclusion

Combined VUV and UVC irradiation represent a promising technology for disinfection and removal of contaminants from water. The in situ formation of radicals may overcome the problem of many UV-based photocatalytic methods in which the residual catalyst needs to be removed from the system, thereby decreasing the cost of the process. Simultaneous irradiation with UVC and VUV light allows the direct and indirect photolysis of chemicals via direct absorption of light and indirect oxidation of the chemical by the action of reactive species generated in the water. In addition, these mechanisms will also contribute to the disinfection of the irradiated water (Wang et al. 2010). Hence, VUV/UVC treatment can be an alternative to other AOP technologies for the removal of a range of contaminants from water. The current study provided a profile of combined VUV/UVC-mediated degradation of TEB in drinking water including occurrence of transformation products and changes in toxicity. The non-invasive VUV/UVC treatment efficiently removed TEB from drinking water and decreased the overall toxicity to a battery of test organisms due to formation of less toxic transformation products. The efficient removal of TEB and the abatement of toxicity suggested that combined VUV/UVC treatment could be a relevant technology for removal of azole fungicides from drinking water.

## Supplementary Information

Below is the link to the electronic supplementary material.

Supplementary file1 (PDF 121 KB)

## Data Availability

The datasets used during the current study are available from the corresponding author upon request.
