# Supplementary material for: Attenuation of toxicity and occurrence of degradation products of the fungicide tebuconazole after combined vacuum UV and UVC treatment of drinking water
Source: Environ Sci Pollut Res Int. 2022 Apr 2;29(38):58312–25. doi: 10.1007/s11356-022-19691-0 (PMC9395489; doi:10.1007/s11356-022-19691-0)
Supplement: Supplementary file 1 — Supplementary file1 (PDF 121 KB) [file 11356_2022_19691_MOESM1_ESM.pdf]

Supplementary material

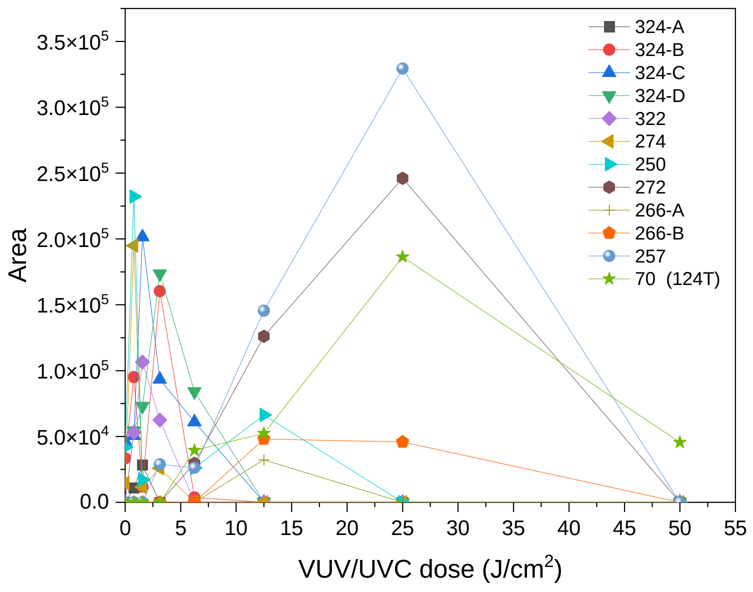

Fig. S1 Transformation products of aqueous TEB samples identified by LC-HRMS after VUV irradiation. Transformation products are denoted by their mass-to-charge (*m/z*) ratio. 124T refers to 1,2,4-triazole.
